# Developing a guideline to standardize the citation of bioresources in journal articles (CoBRA)

**DOI:** 10.1186/s12916-015-0266-y

**Published:** 2015-02-17

**Authors:** Elena Bravo, Alessia Calzolari, Paola De Castro, Laurence Mabile, Federica Napolitani, Anna Maria Rossi, Anne Cambon-Thomsen

**Affiliations:** Department of Hematology, Oncology and Molecular Medicine, Istituto Superiore di Sanità, Viale Regina Elena 299, 00161 Rome, Italy; UMR U 1027, Inserm, Université Toulouse III - Paul Sabatier, 37 allées Jules Guesde F-31000, Toulouse, France

**Keywords:** Biobanks, Bioresource, Bioresource Research Impact Factor, CoBRA, Data sharing, Guideline, Open policies, Repository, Standardized citation, Traceability

## Abstract

**Background:**

Many biomedical publications refer to data obtained from collections of biosamples. Sharing such bioresources (biological samples, data, and databases) is paramount for the present governance of research. Recognition of the effort involved in generating, maintaining, and sharing high quality bioresources is poorly organized, which does not encourage sharing. At publication level, the recognition of such resources is often neglected and/or highly heterogeneous. This is a true handicap for the traceability of bioresource use. The aim of this article is to propose, for the first time, a guideline for reporting bioresource use in research articles, named CoBRA: Citation of BioResources in journal Articles.

**Methods:**

As standards for citing bioresources are still lacking, the members of the journal editors subgroup of the Bioresource Research Impact Factor (BRIF) initiative developed a standardized and appropriate citation scheme for such resources by informing stakeholders about the subject and raising awareness among scientists and in science editors’ networks, mapping this topic among other relevant initiatives, promoting actions addressed to stakeholders, launching surveys, and organizing focused workshops.

**Results:**

The European Association of Science Editors has adopted BRIF’s suggestion to incorporate statements on biobanks in the Methods section of their guidelines. The BRIF subgroup agreed upon a proposed citation system: each individual bioresource that is used to perform a study and that is mentioned in the Methods section should be cited as an individual “reference [BIORESOURCE]” according to a delineated format. The EQUATOR (Enhancing the QUAlity and Transparency Of health Research) network mentioned the proposed reporting guideline in their “guidelines under development” section.

**Conclusions:**

Evaluating bioresources’ use and impact requires that publications accurately cite such resources. Adopting the standard citation scheme described here will improve the quality of bioresource reporting and will allow their traceability in scientific publications, thus increasing the recognition of bioresources’ value and relevance to research.

Please see related article: http://dx.doi.org/10.1186/s12916-015-0284-9.

## Background

### Sharing bioresources: needs and impediments

Bioresources are collections of data and/or samples that are scientifically built and systematically documented. These include physical resources like human biobanks with associated health information, databases, plant and animal repositories, registries, and bioinformatics tools. Although an increasing proportion of biomedical research relies on bioresources, these are seldom shared [1]. In clinical research, for instance, nearly half of trials remain unpublished [2]. In some instances, data sharing may be prohibited to protect subject/patient/victim confidentiality, proprietary interests or national security, or for political reasons, but these are very specific cases. Most of the time, sharing does not occur because it entails a long and time-consuming process that is far from fully appreciated and which is hardly recognized by stakeholders.

Sharing bioresources does not simply involve providing access to other users. Good sharing requires specific work on metadata, on quality control for data and samples management, and on documentation. Sharing also requires regular bioresource updating and the development of a clear access policy. This work is not trivial. It is neither recognized and valued in the present academic world nor considered an important aspect in the processes of evaluation. This lack of recognition is a major obstacle to sharing. The more difficult, costly, time-consuming, and highly specialized the development of a useful bioresource is, the more researchers and institutions will expect recognition for their usefulness. If the scientific community does not value this work, it is unlikely to be performed adequately.

Sharing research outputs, data, and resources contributes to building reliable knowledge and generating innovation. This is now supported by funding agencies that foster data sharing, especially in health research [3]. The European Commission emphasizes that research data are as important as publications, and it has committed to openness in its present funding scheme, Horizon 2020 [4]. A number of initiatives are being developed to encourage exploiting existing healthcare databases and exchanges in the medical world [5]. Initiatives of interest include the Canadian Network for Observational Drug Effect Studies [6] and the initiative for Quality Assessment of Administrative Data [7] of the Institute for Clinical Evaluative Sciences. Some journals, including Annals of Internal Medicine [8], provide guidance on data sharing and a number of authors have published articles addressing the issue of sharing health data [9,10].

However, these kinds of initiatives mainly concern patient/trial data, and no standard exists regarding the use of bioresources in general [1,11,12]. One potential solution, outlined in this article, is to develop a way to incentivize the biomedical community through harmonized citation and recognition processes.

### Why do we need a standard for bioresource citation?

As explored in a previous article [1], it is extremely difficult to identify the contribution of any specific bioresource to research published in scientific articles because bioresources are either cited in a confusing, heterogeneous, and unstandardized way, or they are not cited at all.

In most cases, due to the current modalities of citation, the use of a bioresource in a research article (summarized below) is not retrievable via PubMed or other bibliographic databases, which only index abstracts. This does not allow proper traceability and visibility of bioresources in scientific literature or in other (online) sources that would highlight their use and thus encourage bioresource sharing. For instance, an analysis published in 2011 showed that half of the papers published in biomarkers research contained no information about the biospecimens used [13].

The points below summarize some of the negative effects of citation heterogeneity on the tracking of bioresource use, and the limitations generated by this situation. Adopting a citation standard would bring several advantages, which are also outlined.

*Current modes of bioresource citation*

- Bioresources may be acknowledged in various sections, including Materials and Methods, Acknowledgements, and References
- Bioresource acknowledgements or citations may be placed outside the main paper, or in online supplementary materials
- Citations may acknowledge different resource levels, for example, citation of the consortia or network, but not the individual bioresource
- Secondary use, such as the use of derivatives from the original bioresource (i.e., extracts from biospecimens)
- Typing errors or approximation of the bioresource name/identification; a multiplicity of names for a given bioresource; various names in different languages
- Acknowledgement of persons and authorship instead of the bioresource itself
- Absence of bioresource citation (negligence)
- Websites: reference to web sites that no longer exist, are not updated, or are not informative
- Absence of a Material Transfer Agreement or Data Transfer Agreement number and/or report information on the access(es)

*Effects of variations in bioresource citations on tracking bioresource use*

- Full-text mining is required to trace bioresource use
- No traceability
- Difficult or impossible to recognize the use and contribution of individual bioresources
- Disregarded criteria for correct reporting
- At worst, no tracking is possible if there is no citation at all
- Misleading or incomplete information about bioresource use

*Effect of the lack of standardized citations*

- Bioresources are not indexed in PubMed or Web of Science
- Incomplete information of the bioresources used in research articles
- Lack of recognition and traceability
- Studies may be impossible to replicate by others
- No information on the number and type of biosamples, the amount of associated data, or the data exchanged between the biobank and the user
- Limited ability to develop accurate indicators of bioresource activity
- Limited ability to adopt suitable policy for stakeholders at any level
- Underestimation of the utility of bioresources

*Advantages of standardized citation*

- The possibility to search literature for the use of a bioresource
- Available information about bioresource use
- Development of indexing tools in PubMed and Web of Science to track the use of bioresources
- Support for the development of metrics to assess bioresource impact
- Facilitation of stakeholders’ work (e.g., policy, decision-making, assessment, etc.)
- Increased recognition of infrastructure and institutions involved in creating and maintaining a bioresource
- Improved specific knowledge of biospecimens and databases used in research articles
- Increased sharing of data and biological samples
- Improved trust of bioresource contributors including patients and donors

To track publications involving a bioresource, it is essential that researchers consistently acknowledge the use of the bioresource by placing unique and traceable information in all relevant publications in a defined section of the article. Ideally, this information should include an actionable digital identifier (ID) assigned to the bioresource. To date, such an ID is not available. In order to fulfil the requirements of the scholarly record, a bioresource ID should be persistent, globally unique, citable, and easily retrievable through the Internet. There is major debate over which body or bodies should be responsible for assigning and managing bioresource IDs [11].

The systematic and standardized citation of bioresources in journal articles is needed for the fair recognition of the impact of bioresources on health research, both in qualitative and quantitative terms. While textual resource citation follows clear editorial guidelines, citation rules for bioresources are yet to be defined. Most current initiatives address data sharing policies or technical aspects of sharing, such as interoperability, quality of data, and standards for data management, but not the recognition of the work required in data sharing and the traceability of such sharing [1,14,15]. However, a recent initiative by publishers has begun to address this issue [16]. The Bioresource Research Impact Factor (BRIF) initiative has taken the lead on activities aimed toward standardizing the bioresource citation process, together with biomedical editors [17,18] and other scientific organizations involved in bioresource monitoring and sharing. This process of collaboration and its principal outcomes will be discussed in detail in the following paragraphs.

### The BRIF initiative

The BRIF initiative [19] is an on-going international framework, the major features of which are summarized in Table 1. Five dedicated working subgroups deal with priority tasks: adapting ways of identifying bioresources, parameters to consider when calculating impact, standardized ways of citing bioresources in scientific literature, the embedding of BRIF in data sharing policies, and dissemination. The “BRIF and Journal Editors” subgroup first established connections with editors to standardize bioresource citation. The next phase is to implement BRIF, once it has been assessed, with a specific task being to assess proposals for amending available editorial guidelines. The subgroup gathers three editorial experts and two biomedical researchers involved in biobanking based at the National Institute of Health (Istituto Superiore di Sanità) in Italy, as well as the BRIF leader and manager, who are from a joint research unit between the French National Institute for Health and Medical Research (Inserm) and the University Toulouse III. All members of the workgroup are involved in coordinating the work carried out with journal editors and in leading the development of a guideline for bioresource citation in the scientific literature.

**Table 1:** **The BRIF initiative** [1,12,19]

**Organization and multidisciplinary competences**	
The BRIF initiative is developing a framework to recognize and measure the use and impact of biological resources in health research. The BRIF working group consists of 135 members from 22 countries, most of whom are either European (86) or North-American (31). A broad range of experts are participating, including biobankers, clinicians, genomic/genetic scientists, epidemiologists, computer scientists, jurists, lawyers, ethicists, experts in information, bibliometricists, and journal editors; they are represented in different BRIF subgroups.	
*General aims*	• Promoting the sharing of data and biological samples
	• Recognizing the work (human resources/infrastructure) involved in setting up and maintaining a valid bioresource
	• Providing more complete information on the bioresources used in research
*Strategies*	• Standardizing the citation of bioresources in scientific articles in order to trace their use on the web
	• Creating a tool (BRIF indicator) to establish the frequency of a bioresource’s use and evaluate its impact based on quantitative metrics and on the use of a unique digital resource identifier (ID)
**Working subgroups**	
• *BRIF and digital identifiers*	Exploring and assessing existing and emerging technical solutions suitable for bioresource identification, as well as addressing key related questions, including what to identify (biobank projects, sample collections, databases, datasets, registries) and which body or bodies should be responsible for assigning bioresource IDs
• *BRIF parameters*	Identifying potential factors to take into account when calculating the BRIF indicator
• *BRIF in sharing policies*	Developing an appropriate set of recommendations (BRIF procedure) to consider in developing and implementing bioresource access and sharing policies
• *BRIF and Journal editors*	Relating with scientific journal editors to standardize bioresource citation in journal articles and amending the corresponding editorial guidelines
• *BRIF dissemination*	Communication and dissemination of information related to the BRIF initiative
**Pilot projects**	
To support the adoption of bioresource citation standards, BRIF participates in two pilot projects:	
• The creation of the open access journal Open Journal of Bioresources [20], which publishes peer reviewed articles (‘marker papers’) that describe bioresources in detail according to a standardized template. The Digital Object Identifier of the marker paper can identify the bioresource and provide a way of automatically tracking the use of bioresources in academic literature	
• Testing the use of a unique bioresource ID in the research community, attributed through the Public Population Project in Genomics and Society [21] in partnership with the European project BioSHaRE (Biobank Standardisation and Harmonisation for Research Excellence in the European Union) [22].	

## Steps

### Developing a guideline for bioresource citation (CoBRA)

The actions of the “BRIF and Journal editors” subgroup were divided into two phases. The first phase aimed at disseminating information about the BRIF initiative among the scientific community and attracting the attention of science journal editors, thus creating or raising awareness about proper reporting and sharing of bioresources. The second phase focused on the practical development of a standard for the “Citation of BioResources in journal Articles” (CoBRA). The structure of the guideline was developed according to the general principles reported by Moher et al. [23].

### Disseminating information on BRIF to science editors

In 2012, the “BRIF and Journal editors” subgroup agreed to submit proposals to international associations, editorial committees, and other professional organizations, with the aim of attracting their attention on issues related to bioresources, creating awareness of the BRIF initiative, and eventually of amending editorial guidelines by including a reference to bioresources.

First, the subgroup contacted the International Committee of Medical Journal Editors (ICMJE), a group of general medical editors and representatives of related organizations who evaluate how to improve the quality of medical science and reporting by updating their recommendations [24]. The aim of this action was to suggest amendments concerning standardized bioresource citations to be included in a revised version of the ICMJE Recommendations for the Conduct, Reporting, Editing, and Publication of Scholarly Work in Medical Journals (at that time named Uniform Requirements for Manuscripts Submitted to Biomedical Journals [25]). The subgroup thoroughly analyzed the ICMJE recommendations and suggested specific amendments.

Second, the subgroup examined the Guidelines for Authors and Translators of Scientific Articles produced by the European Association of Science Editors (EASE), an internationally oriented community of individuals from diverse backgrounds, linguistic traditions, and professional experience who are interested in science communication and editing. EASE guidelines are well known by science editors not only in Europe but also globally, thanks to their translation into 22 languages [26]. The subgroup identified specific sections where these guidelines might include standardized bioresource citations. During the 11th EASE General Assembly and Conference, “Editing in the Digital World,” held in Tallinn in 2012 [27], these proposed amendments were presented and discussed.

Third, the subgroup also approached the Committee on Publication Ethics (COPE), an important forum for editors and publishers of peer reviewed journals, which advises editors on handling cases of research and publication misconduct. The subgroup encouraged COPE to consider the editorial and ethical problems concerning biobanks and bioresources in general.

Fourth, the subgroup contacted the Enhancing the QUAlity and Transparency Of health Research (EQUATOR) Network, which works to improve the reliability and value of medical research literature by promoting transparent and accurate reporting of research studies, in order to discuss how a standard for bioresource citation currently in development could be announced and reported on the EQUATOR website [28].

In pursuing these steps, the subgroup participated in a number of international meetings and events. The preliminary work of disseminating information on BRIF initiatives was achieved through a lecture at the 5th Belgrade International Open Access Conference in 2012 [29] and a poster at the 11th EASE General Assembly and Conference, described above [27]. Given the interest generated, the subgroup targeted other potentially interested key communities by participating in their international meetings and events. In the science editing community, this included:

- The Seventh International Congress on Peer Review and Biomedical Publication, 2013, Chicago, USA;
- The Eurosurveillance Editorial Board Meeting, 2013, Vilnius, Lithuania;
- The 12th EASE General Assembly and Conference, 2014, Split, Croatia;
- The 14th European Association for Health Information and Libraries Conference, 2014, Rome, Italy.

In addition, the subgroup presented the BRIF initiative in scientific conferences attended by scientists who work with large databases and/or collections, including:

- The European Human Genetics Conference, 2013, Paris, France;
- The Brocher Workshop Exploring Innovative Mechanisms to Build Trust in Human Health Research Biobanking, 2013, Geneva, Switzerland;
- The “HandsOn: Biobanks 2013” Conference, The Hague, Netherlands;
- The 28th European Immunogenetics and Histocompatibility Conference, 2014, Stockholm, Sweden;
- The 5th Biennial Meeting of the Human Variome Project, UNESCO, 2014, Paris, France;
- The “HandsOn: Biobanks 2014” Conference, Helsinki, Finland;
- The ESBB (European, Middle Eastern and African Society for Biopreservation and Biobanking) annual meeting, 2014, Leipzig, Germany.

Informal contacts and discussions occurred during these events, increasing the dissemination of information. The topic was always well received, and the subgroup was encouraged to continue.

### Developing a guideline for bioresource citations

In 2013, based on the experiences above, the “BRIF and Journal editors” subgroup decided to further explore how clear bioresource citation guidelines could be established with the help of journal editors. A survey was presented to a sample of biomedical editors in order to identify key science editors who would be interested in the initiative and who would contribute to its advancement. In order to test the feasibility of the approach, the survey was addressed initially to 50 editors of journals, both open access and traditional subscription-based, that are indexed in the Web of Science and that have a range of impact factors. Subsequently, to diversify the sample, a second group of 40 editors were contacted, identified according to the same criteria. The survey response rates were 22% and 17.5% for the first and second groups, respectively.

The survey questions were as follows:

- Have you ever heard about the issues of bioresources evaluation and their standardized citations in biomedical journals?
- Do you think this is a topic of interest for the editorial policy of your journal?
- Would you agree to address this issue in the Instructions to Authors?
- A meeting will be held in Rome (June 21, 2013) to discuss about this matter. Would you like to receive more information?
- Would you consider participating in this meeting?

Interested editors identified by the survey were invited to participate in a workshop organized in Rome, Italy, in June 2013.

At the Rome meeting [30], participants were split into two working groups selected to balance individual competences and to facilitate individual contributions to the debate. Participants were asked to discuss proposals including the following as potential recommendations to authors in journal guidelines:

- A unique identifier for each biobank – or bioresource – that could be used as a form of citation;
- A dedicated “bioresource section or field,” analogous to the existing “author section” or “author field” that authors are required to complete during the article submission process, which would require authors to include specific metadata characterizing the bioresource used and thereby allowing its easy tracing;
- The use of specific required phrases to acknowledge bioresource(s).

## Results

### Engaging the science editing network

EASE agreed to incorporate statements on biobanks in the “Methods” section of the EASE Guidelines published in 22 languages [26]. The answers from ICMJE and from COPE oriented the “BRIF and Journal editors” subgroup action toward EQUATOR. According to EQUATOR’s positive evaluation of a standard for reporting bioresource use, the subgroup decided first to develop the standard before contacting other key organizations such as the World Association of Medical Editors, the Council of Science Editors, and the Board of Editors in Life Sciences.

### Developing a standard for bioresource citation

The discussion at the 2013 BRIF workshop in Rome focused the subgroup on a new and practical approach to the issue of bioresource citation. In this sense, the meeting was a turning point. Initially, the main objective of the meeting was to develop a consensus recommendation for standardized bioresource citation. Three leading proposals for bioresource citations in journal articles were presented to the two workshop groups, as discussed above:

1. The use of a unique ID for bioresources;
2. The possibility of a “bioresource field” as editorial metadata;
3. The use of specific acknowledgement sentences.

A subsequent general discussion combined and harmonized the final remarks elaborated by the two groups. The discussion was very lively, several issues were examined, and new proposals were suggested.

The editors shared their points of view about integrating new editorial elements regarding bioresources and stressed how difficult it is to satisfy each stakeholder in journal articles, including authors, readers, resource providers, and editors. They agreed that bioresources should be cited in a standardized/harmonized way, favoring the easiest implementation routes such as using editorial processes familiar to the scientific community and pre-existing solutions that are easy to adapt to the specific case of bioresources.

Regarding the three proposals under discussion, the participating editors agreed that:

- Identifying bioresources using an ID rather than the name of the bioresource would be better to avoid confusion, but that such an ID is not yet generally available;
- A “bioresource field” or a specific section is not an appropriate solution, because it would be too complicated and not of sufficiently broad use to warrant its creation;
- Referring to bioresources in the Methods section seemed the most appropriate solution, provided that the reference corresponds to a cited reference (see below);
- Specific sentences in the Acknowledgement section in journal articles did not seem useful, considering the necessity of traceability and easy retrieval.

The work and discussion among the editors and the scientists created a new paradigm that takes into account standardization and traceability by adopting the simplest solutions among currently existing options. The core idea is to cite each bioresource used in a research work as a reference. To identify the bioresource in the reference, an immediate solution would be to use an ID such as a Digital Object Identifier (DOI) corresponding to the publication that describes the bioresource in detail (marker paper), or the bioresource catalogue ID. Initiatives such as the *Open Journal of Bioresources* [20] or the *Biopreservation and Biobanking* journal [31] that provide bioresource descriptions were determined to be suitable tools for referring to bioresources, although not all bioresources are currently described in corresponding marker papers. The bioresource identifier could then be tracked through CrossRef [32]. According to this proposal, the “Reference” section in the journal article is responsible for the actual task of bioresource citation. This proposal has the advantage of being familiar to researchers, editors, and publishers.

The editors attending this meeting also provided suggestions about where exactly to cite bioresources in journal articles and recommended that guidelines to authors should be revised accordingly. Given the lack of guidelines specifically related to bioresource citation, and following the suggestion of the ICMJE, the “BRIF Journal editors” subgroup approached the EQUATOR Network and submitted the guideline proposal for EQUATOR’s “Reporting Guidelines under Development” section. The request was accepted and the project of a guideline was posted in October 2013 [28].

The proposal for the specific reference scheme to report the use of bioresources was presented and discussed during the Seventh International Congress on Peer Review and Biomedical Publication in September 2013 [33]. This was an opportunity to discuss the issue with the biomedical journal editors, in particular with those involved in the reporting of clinical trials, following which began the elaboration of a specific guideline for standardized bioresource citation. Dissemination of such a proposal was treated as a continuous process aimed at collecting feedback from interested communities to develop the guideline adequately up until its endorsement. The resultant consensus, spanning many different scientific contexts, regarding the information to be included in research studies that use bioresources persuaded the subgroup to propose a guideline on how this information should be reported in scientific papers.

### Guideline for the standardized citation of bioresources in journal articles (CoBRA)

Each bioresource cited in the Methods section of a research article must refer to an individual reference that follows a specific format as described below. The checklist for standardized bioresource citation is reported in Table 2 with further explanation.

**Table 2:** CoBRA checklist for the citation of bioresources used* in scientific journal articles

**Article text section**	**Item #**	**Guidance**	**Additional information**
**Abstract**	1	Indicate whether the work has used one or more bioresources; specify the number of bioresources if relevant	Adapt according to the number of words allowed
**Introduction**	2	Indicate that the work has used one or more bioresources; specify the type	The types of bioresources include: data, samples and data, database, registry
**Methods**	3	Report each individual bioresource used to perform the study:	The format of the reference is detailed in item 6 in the section “References”
		By their name and other ID, if extant	The bioresource name should be the original name as reported in
		By a single bibliographic reference	Official documents such as Material Transfer Agreement (MTA) and Data Transfer Agreement (DTA); the name should be reported in the original language without translation
			Specify any relevant characteristics of the bioresource, such as sample number and type of biospecimens, if this information is not available from the bioresource reference
			Number of accesses can be also reported here, for instance, as the MTA/DTA registration number associated with each access; if the dates of actual bioresource availability for the user (e.g., reception of samples) are distant from those in the MTA signature, this can be reported here
**Results**	4		Indicate the relevance of the bioresource(s) used for the study (Optional)
**Discussion**	5		Standard rules should apply
**Reference**	6	Cite each bioresource as a reference as follows:	Each citation includes three fields: Identification/Institution/Access
		ID/Bioresource Name (acronym if available)/organization or network partnership, membership (optional)/Number of access(es), Date of last access; [BIORESOURCE] Specifications for ID: Unique ID can be DOI, catalogue number, or the name only If the only ID is the name then add Town and Country	The “use” of the bioresource is distinguished within the citation by adding “[BIORESOURCE]” at the end of the reference
			**ID:** citing the ID, rather than, or in addition to, the name is essential in order to avoid any confusion and facilitate retrieval (see Box 1, Example 1)
			**DOI:** if the detailed description of the bioresource is available in a marker paper, it should be cited here, this being one way of providing a DOI (see Box 1, Example 2)
			**Name:** the name should be the original name as reported in official documents such as MTA and DTA. The name should be reported in the original language of the residence country without translation
			**Place of residence** (town) and country should be translated in the article language (See Box 1, Example 3)
			**Acronym:** when available, stable and consolidated, it is recommended to add the acronym to the reference (See Box 1, Example 3)
			If the bioresource requires mentioning **membership or partnership** in consortia, networks or organizations, a dedicated field should be included (see Box 1, Examples 1 to 3)
			When the bioresource is a physical resource such as a biobank or collection, the number of accesses should be specified, in addition to the date of last access. These data will generally correspond to the data signature of the MTA/DTA
			When the bioresource is a digital resource such as a database, dataset, or registry, only the last access should be reported (see Box 1, Example 5)
**Authorship**	7		Standard rules should apply
			Providing samples or data is not sufficient to justify authorship
**Acknowledgements**	8		Standard rules should apply

*In the case of bioresources not used as a source of material for the study, but only referred to, follow the citation format: ID/Bioresource Name (acronym if available)/organization or network partnership, membership (optional) (see Box 1, Example 5).

Examples of the different cases for bioresource citation are given in Box 1.

Bioresources may be cited in a scientific article for two reasons:

- **USE:** The bioresource has been used as a source of samples and/or data to perform the study;
- **NO-USE:** It is necessary to cite a bioresource in the article, but the bioresource was not a source of materials for the study.

In both cases, the “citation” of the bioresource will be implemented following CoBRA indications, but the “use” of the bioresource in the reported study will be specified within the citation by adding “[BIORESOURCE]” at the end of the reference.

**USE: Citing a used bioresource.** The reference has the following format:

**Identification (ID and/or DOI,** Bioresource name, Acronym (if available); (if applicable) Organization (network partnership or membership); **Number (No) of access(es)/Date of last access;** [BIORESOURCE].

**NO-USE: Citing a bioresource that was not used.** The reference has the following format:

**Identification (ID and/or DOI,** Bioresource name, Acronym if available); (if applicable) Organization (network partnership or membership).

An example of these different uses is provided in Box 1, Example 5. If the bioresource used is a digital resource (for example, a database, dataset, or registry) only the last access should be reported. For the sections authorship, acknowledgements, and/or editorial policies, the authors should refer to the instructions for authors of the journal.

### Expected impact of the adoption of CoBRA guidelines

The driving principle of this work is that bioresources must be cited in a standardized and retrievable way in order to acknowledge their value and highlight their use in research achievements. The proposed guidelines were formulated with extensive stakeholder involvement and through a long process of consultation and dissemination, as described. These interactions raised awareness and created consensus on the importance of standardized citations as a tool to measure the impact of bioresources on research production. The main advantages of the adoption of standardized citations are summarized in the *Advantages of standardized citation* section above.

The identification of bioresources through a unique digital ID should be preferred. However, due to the absence of governing bodies in charge of assigning and managing bioresource IDs, few bioresources currently have such an ID. This may soon change, at least in Europe. The principle of assigning a unique ID to biobanks has been adopted by the Biobanking and Biomolecular Resources Research Infrastructure (BBMRI), which was officially awarded the community legal framework for a European Research Infrastructure Consortium (ERIC) on the 3rd of December, 2013 [34]. The mission of BBMRI-ERIC [35] is to facilitate access to and use of high quality human biological resources. Thus far, 12 European countries have already signed agreements to participate, and more countries are expected to join in 2015. An ERIC institution in biobanking provides relevant new perspectives because ERIC is an internationally recognized legal entity that has operational sites (“National Nodes”) in multiple countries. ERIC institutions operate under unique legislation, and the long-term economic sustainability of the consortium is ensured by member states’ annual fees [36].

At present, an increasing number of bioresources publish a corresponding paper that describes the bioresource in detail. In the absence of a unique ID for the resource itself, the DOI corresponding to this descriptive publication may be used as identifier (see Box 1, Example 2).

Identification of a bioresource though a digital identifier (ID/DOI) instead of its name will improve web tracking because i) it allows the standardization of the bioresource tracking process and the use of automated systems, and ii) it enables bioresources to be tracked through CrossRef [32].

Bioresources may partner with networks or consortia and/or they may be members of organizations. Bioresources should mention such partnerships in scientific publications. The reference format proposed allows this (see Box 1, Examples 2 and 3).

BRIF’s concept and the effort to standardize bioresource citation are in line with the multiple initiatives that support open access to research results. A proper bioresource citation system would foster a climate of trust and transparency among all parties involved in research biobanking, from patients to funding bodies and policymakers.

The European Commission policy encourages data sharing and reuse. This policy is at the basis of the recently constituted European BBMRI-ERIC [35], which is including several axes of the BRIF initiative in its work plan – a major step in BRIF implementation. It is necessary to implement systematic reward and recognition mechanisms for sharing data and materials. The sharing of, or reuse of stored biological samples and associated research data has become key to developing scientific knowledge. Consistently and easily citing sources facilitates their recognition and makes their impact measurable, as with traditional manuscript publications. The standardized citation proposed in this guideline allows the efficient retrieval of the bioresources used in journal articles. It is a “ready to use” tool for beginning to measure bioresource impact.

In human health studies, bioresource use is at the base of translational research development. The adoption of standardized bioresource citations and the BRIF initiative (Table 1) is in harmony with the needs of public health systems to improve quality and to develop cost affordable indicators of outcomes [37]. The implementation of a citation schema also has the advantage of not creating any additional cost for researchers and stakeholders. Initiatives such as The Biospecimen Reporting for Improved Study Quality, which are recommendations intended to improve the quality of research in which human biospecimens are used, are very useful [38] and the CoBRA guideline proposed for citations is complementary to them.

The authors expect that the CoBRA guideline will be published in the reporting guidelines of the key website EQUATOR [28]. In fact, the CoBRA standard is consistent with all of the guidelines currently published in EQUATOR and can be used in combination with any of them. For example, a randomized trial involving the use of a bioresource should use the CONSORT guideline [39] in order to decide which information to include about the trial (including trial design, participants, sample size, randomization, and so on) and the CoBRA guideline in order to properly cite the bioresource(s) used according to the standard format.

## Conclusions

Evaluation of bioresource use and impact in research requires accurate citation of such resources in publications. Editors, as gatekeepers of scientific information, and authors as providers of such information, must be proactive in applying CoBRA as a standard citation scheme for bioresources. The use of CoBRA will improve the quality of bioresource reporting and will allow bioresource traceability in scientific publications, encouraging policies of collaboration and sharing. The endorsement and the adoption of the CoBRA guideline by authors, editors, researchers, and bioresource policy stakeholders is the first necessary step to achieve these goals and is essential to enhance transparency in health research.

## Abbreviations

BBMRI: Biobanking and Biomolecular Resources Research Infrastructure
BRIF: Bioresource research impact factor
CoBRA: Citation of BioResources in journal Articles
COPE: Committee on Publication Ethics
DOI: Digital object identifier
EASE: European Association of Science Editors
EQUATOR: Enhancing the QUAlity and Transparency Of health Research
ERIC: European research infrastructure consortium
ICMJE: International Committee of Medical Journal Editors
ID: Digital identifier
